# The art of Patient and Public Involvement: exploring ways to research and reduce air pollution through art-based community workshops – a reflective paper

**DOI:** 10.12688/wellcomeopenres.17886.1

**Published:** 2022-05-26

**Authors:** Shahid Islam, Rukhsana Rashid, Maria Bryant, Holly Schofield, Rosemary R.C. McEachan

**Affiliations:** 1 ActEarly Consortium, Bradford Institute for Health Research, BRADFORD, West Yorkshire, BD9 6DA, UK; 2 Born in Bradford, Bradford Institute for Health Research, BRADFORD, West Yorkshire, BD9 6DA, UK; 3 Department of Health Sciences and the Hull York Medical School, University of York, York, Yorkshire, UK; 4 Leeds Institute of Clinical Trials Research, University of Leeds, Leeds, West Yorkshire, LS2 9JT, UK

**Keywords:** Air pollution, Art, Patient and Public Involvement, Wittgenstein, Clean air zone

## Abstract

In this reflective paper we outline and discuss our art-based Patient and Public Involvement (PPI) approach.  This exercise held two broad objectives. Firstly, to assist policy makers in understanding the types of interventions communities will find acceptable to address the problem of poor air quality, and secondly, to ascertain community views about our research plans to explore the impact of the planned interventions on neighbourhoods.  We reflect on both our approach and the emergent conversations from the PPI activity.

Attendees contributed to the process and stressed the importance of not burdening poor neighbourhoods with costly charges as that would ameliorate one health problem but generate others as a consequence of additional financial burden.  Equally, they stressed the need to conduct research on matters which they could connect with such as the impact of clean air plans on young children and how information about air pollution is disseminated in their neighbourhoods as and when research findings emerge.

This paper offers a conceptual analysis of the art-based PPI method and uniquely draws a connection to the philosophical traditions of Ludwig Wittgenstein.  Specifically, we demonstrate how art is conducive to creating a dialogue which is specifically helpful for PPI purposes for both researchers and implementers, and conversely, why traditional conversational approaches may have fallen short of the adequacy mark in this regard.

## Disclaimer

The views expressed in this article are those of the author(s). Publication in Wellcome Open Research does not imply endorsement by Wellcome.

## Background on air pollution

A recent study highlighted why air pollution should be treated as a pressing public health priority in the UK after it had been attributed to causing 64,000 premature deaths every year ^1^. Exposure to poor air quality has been linked with a range of health problems including poor birth outcomes ^2^, cardiovascular events and mortality ^3^, respiratory illness ^4^, lung cancer ^5^, cognitive development and neurological disorders ^6^ and pre-term and low weight birth ^7^.

Evidence shows that air pollution is often higher in more deprived communities ^8– 10^ and that high mortality rates strongly correlate with socio-economic deprivation. This is further compounded by neighbourhood design and demographics, as areas exposed to higher amounts of air pollution also contain the fewest green spaces and have higher levels of population density ^11, 12^. All these variables coalesce and compound existing health inequalities with young children considered a particular risk group, as data shows 1 in 3 babies are born in areas of the UK with dangerously unsafe levels of traffic related pollution ^8, 13^. In Bradford, health data and research findings identify that up to 33% of childhood asthma cases are linked to poor air quality ^14^ and excessive levels of pollution across the city account for a multiple number of morbidities ^12^.

Bradford’s Local Authority received a directive from the UK central government to create a plan to reduce pollution to legal levels in ‘as quick a time as possible’ after levels of air quality were found to be in breach of EU legal limits ^15^.The key intervention to achieve a reduction in air pollution is the introduction of a charging clean air zone (CAZ) ^13^. A charging clean air zone involves charging older, more polluting vehicles (petrol vehicles below the Euro 4 standard implemented in 2005, and diesel vehicles below the Euro 6 standard implemented in 2014) a daily levy to enter the zone. The Government directly asked Bradford’s Local Authority to consider implementing one of four ‘classes’ of clean air zone, ranging from less stringent (Class A: only charging non-compliant buses, coaches and taxis) to the most stringent (Class D: charging buses, coaches, taxis, heavy goods vehicles, light goods vehicles, and private vehicles). 

## Why community voices matter

Whilst planners and implementers may be confident about the causal relationship between pollution from motor vehicles and ill health; evidence shows us that “ *knowing the prevalence and causes of a health problem does not always tell us the most effective way to reduce it*” ^16^ (p.5) or even how acceptable any planned interventions will be to the target community. Our research, for example, which was informed by, and came after the PPI activity described in this paper, explored levels of acceptability and potential unintended consequences of introducing Clean Air Zones and found that whilst most people are supportive of efforts to clean the ‘invisible air’, this was not without reservations about why this issue should be prioritised over other more ‘visible’ matters ^17^. A number of interviewees in the study placed more weight on tackling, what seemed to them, more pressing concerns such as fly-tipping, dangerous driving and other hazards within their neighbourhoods with lesser emphasis placed on reducing pollution ^17^. 

This example signifies the importance of Patient and Public Involvement (PPI) as a necessary component for research and implementation plans. In particular, why it is necessary to be mindful about the difference in perceptions between community priorities and implementation plans when it comes to addressing any given issue. The National Institute for Health & Clinical Excellence (NICE) ^18^ further reinforce this significance by making the following point about ownership:

*“Communities that identify and articulate what is most important to them, and agree clear aims for the initiative, are more likely to develop a positive relationship with the commissioner, 'own' the initiative and get more benefit from it. Health and wellbeing initiatives that are developed in partnership between local communities and commissioners are more relevant and meaningful to the community” (p.5).*

The government guidance on the CAZ initiative took a similar line: “ *Local knowledge is vital to finding solutions to air quality problems that are suited to the local areas and the communities and businesses affected* ^13^ *(p.7–8)*

## The inherent challenges of PPI

Academic and policy discussion on PPI tend to adopt a positive tone to celebrate the democratic and ethical nature of including people in research plans ^19, 20^, which is often reflected in straplines such as *“nothing about us without us”* ^21^ and “ *from users and choosers to makers and shapers”* ^22^ however, one central challenge which is seldom addressed and often masks this positive zeitgeist remains unresolved. That is - we know ‘why’ PPI is important but the question remains on ‘how’ to deliver it in an effective and inclusive way that is guided by evidence ^23^. There is an implicit assumption at play here which makes PPI appear a straight-forward activity which does not warrant any level of sophistication to achieve the collation of public views. Rigorous and sophisticated designs are usually reserved for the methodology section for tasks such as data collection and analysis as these are considered cornerstones of research activity. PPI, on the other hand, is often seen as a formulaic hurdle which can be reduced to a matter of sharing a research plan with members of the public and simply seeking their feedback about the expressed ideas. In most cases this is delivered as a consultation exercise where people are invited to comment on what is presented before them ^24^. 

An equally noteworthy point to draw attention to here is the problematic nature by which proceedings from PPI activity are recorded and actioned; whilst commendable efforts have been made to generate evidence based guidelines for the reporting of PPI such as those put forward by Staniszewska and colleagues ^23^, without their widespread application, the *status quo* will remain unchanged. In making a case for evidence informed ways to pursue PPI activity, Staniszewska *et al.* ^23^ trenchantly outline the consequences that can follow when PPI methods are not properly reported. As they put it:

*“Inconsistent reporting creates a fragmented evidence base making it difficult to draw together our collective understanding of what works, for whom, why, and in what context. Furthermore, researchers, patients, carers, or clinicians cannot learn from previous experience, and precious resources devoted to involving patients and the public are wasted. Omitting descriptions of PPI activities from a study can represent a form of misreporting and might misrepresent the initial intentions of a study”* ^23^ *(p.2).*

This demonstrates why the enterprise of PPI can seem fraught with uncertainties and inconsistencies. De Wit *et al.* ^25^ in recognising this, place the blame squarely at the doorstep of training and education programmes in health research, which they say, pays limited attention to PPI matters. These authors argue that most research training only pays scant attention to PPI with the following implications as a consequence:

*“PPI is rarely part of the basic research curriculum of PhD candidates, and they (PhD candidates) face several challenges when they want to start engaging patients. They lack knowledge on concepts of PPI and ways of applying them in practice. Not knowing the benefits and pitfalls of different options and their impact on the research outcomes is one of the reasons for not using the most appropriate PPI methods”* ^25^ *(p.753).*

## Our art-based PPI method

In recognition of the common inadequacies of PPI, we set out to design PPI activities using a meaningful and consistent approach to better understand what effect, if any, Bradford community residents felt the CAZ proposals may have on their local neighbourhoods. To this end, we led three art-based community workshops to engage members of the public and elected ward councillors to understand attitudes towards four types of government endorsed clean air zone proposals. We aimed to achieve two outputs from this endeavour; first, to collect and feedback perceptions held by community members about the acceptability of the proposed interventions to the Local Authority; and second, to generate ideas about research questions and topics for the associated emerging research plans about the CAZ. 

## Ethics

The workshops were PPI exercises and so consent for participation and ethical approval were not sought at this developmental stage. Equally, information generated through such exercises are not recorded or utilised in the same way as research data.

## Invitation, attendance and venues

In total, 15 members of the public living in pollution hot-spots and 14 elected ward councillors who represent various constituencies across the city attended three workshops between February to July 2019. The workshop with community members was held at a community venue which is located in the grounds of a popular park without any ward councillors present. The two workshops with the councillors were held at the Town Hall and had no community members (constituents) present.

The members of public were invited through an email sent to people who work in community engagement roles in a variety of voluntary and community-based organisations. We encouraged the email recipients to inform people in their networks about the workshop with a view to encouraging people to attend. We made a special effort to encourage people who are members of resident associations or volunteers in local organisations by phoning them and explaining the purpose of the activity and specifically requesting their attendance. 

The elected ward councillors were invited via their official council email. We then followed this up by asking Local Authority employees, who are responsible for the implementation of the clean air plans, to promote the importance of the PPI exercise to the Councillors in the hope this will raise their interest and improve attendance.

## Workshop content

The workshops were designed to encourage topical discussions with a special focus on dilemmas and challenges that might arise through implementing a range of CAZ options recommended by the UK Government as part of their Clean Air Strategy ^13^. To help us facilitate these discussions, two of the authors met an art illustrator along with a Local Authority representative and provided details on what needs to be sketched for the PPI posters.

## Format

During ‘welcome and arrival’ we asked people to place a red coloured sticky-dot to indicate how much they know about air pollution on a flipchart poster which followed the function of a *Likert* scale with differential levels of awareness placed at each end of the scale (ranging from ‘Very little’ to ‘ A lot’). Attendees were asked to repeat the same exercise, on the same flip-chart, after the event as they were leaving, however this time using a green sticky-dot. This simple and visually engaging task acted as a proxy indicator to show peoples’ knowledge about the subject matter in a ‘before-and-after’ way. 

**Figure 1.**
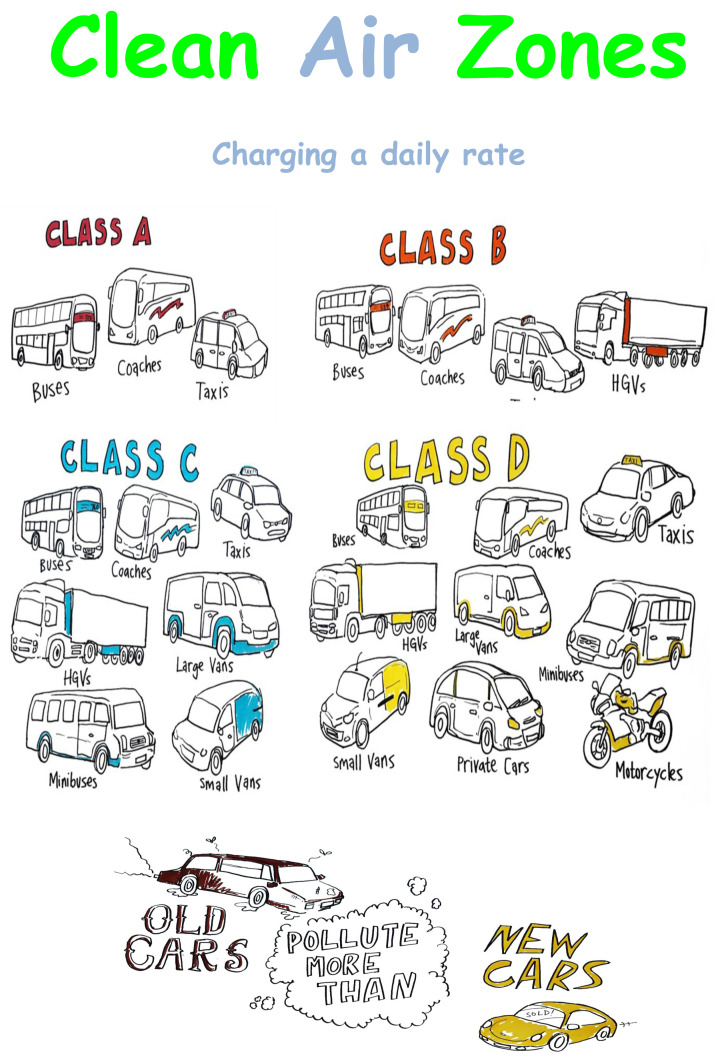
The wall sized poster was split into four types of Clean Air Zones. The key difference between each clean air zone (CAZ) option is each class contains different vehicles with CAZ Class A holding the fewest vehicles and Class D holding the most.

As part of the main PPI exercise, The CAZ illustrated poster ( Figure 1) was displayed on a wall and attendees were asked to hold conversations in small groups about the implications of the different options and from there proceeded to colour, write or sketch their views onto various sections of the poster to reflect how each of the CAZ options might affect their communities. 

**Figure 2.**
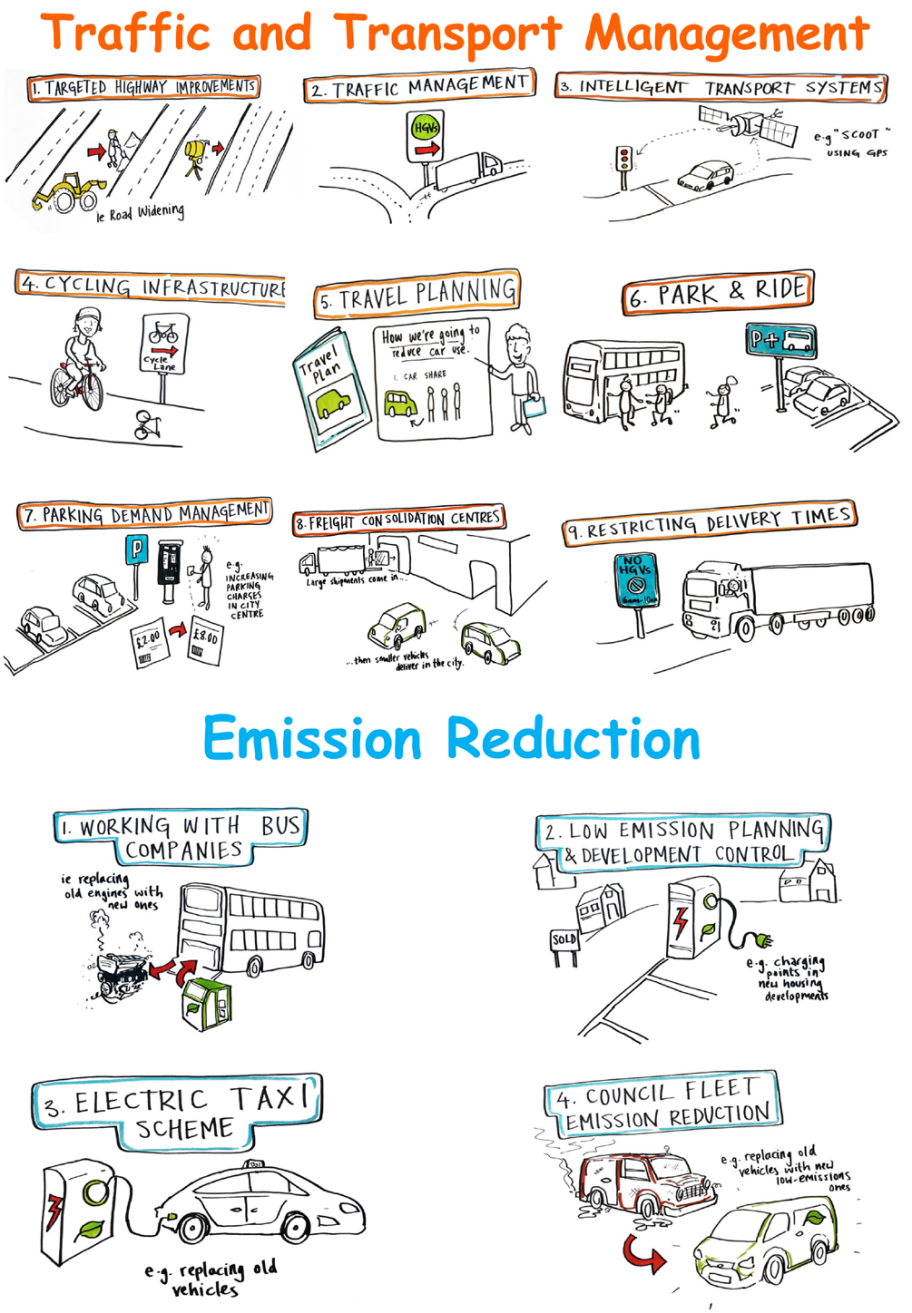
The wall sized poster with supplementary initiatives to tackle air pollution (e.g. improved cycling infrastructure, restricted delivery times and smart traffic lights to reduce congestion etc.).

This same exercise was then repeated for the second poster ( Figure 2), to elicit views about a number of supplementary initiatives which were being considered by the Local Authority to complement CAZ efforts to further reduce air pollution. These initiatives included, amongst others, improving cycling infrastructure and traffic management systems and the introduction of a car park and ride scheme. 

After facilitating the workshops, we collated the key considerations into one short report for the Local Authority, including photographs of the posters after the PPI activity. The report contained pertinent highlights alongside favourable and objectionable comments about each of the CAZ options and the supplementary initiatives. We included suggestions we heard about the research methods, topics and ideas.

## Key highlights from the workshops

Community members and ward councillors immersed themselves into this PPI approach and welcomed plans to improve levels of air quality; however, people were mindful of the consequent economic impact CAZ charges may have on local businesses and residents. This was seen as a difficult *circle-to-square* as attendees acknowledged some action was needed to reduce pollution, but feared that disadvantaged communities, many of which are already under financial strain, may find the charges difficult to absorb and so research needs to be focussed on socio-economic impacts in the local area. 

The workshops highlighted that all CAZ options contain inherent dilemmas which either makes them ‘fraught with complexities’, or run the ‘risk of being ineffectual’ and the research needs to be geared towards understanding what impact the chosen CAZ will have on tackling air quality. For example, attendees felt that Class A CAZ, (where charges would only be limited to non-compliant buses, coaches and taxis), lacked teeth to reduce air pollution as it did not go far enough with what was included. Similarly, when discussing Class D (including charging personal vehicles that did not meet minimum compliance standards), participants saw this as promising in terms of efficacy, but would only work if concerted efforts were made to resolve the issue of unaffordable public transport fares. Attendees repeatedly highlighted these concerns on the posters and then followed up in conversation that driving is always seen as the ‘easy winner’ compared to expensive bus fares particularly as car parking fees were generally seen as low. 

Decisions on modes of travel were equally bounded by how much people knew about air quality and the impact this may have on health. Most of the attendees informed us they had not considered this in any meaningful way until they attended the workshop. One epiphany moment was when attendees in one of the Councillor workshops learnt that passengers inside a car travelling on a busy road were exposed to much higher levels of air pollution (up to 12 times more) compared to people walking or cycling on the side of the road ^26^. The important consideration that stems from this point is the way in which we research and evaluate how people change their behaviour when their knowledge and awareness about air quality increases. 

All of the above points, whilst not research findings in the traditional sense, provided us with important insights on what we should focus on when formulating our qualitative research questions. When asking attendees to directly suggest ideas on what topics we should prioritise for research from a community point of view, a number of suggestions were made including; to fit air quality monitors to pushchairs to measure exposure during the school run and to conduct survey based research asking school children – *how did you get to school and why did they travel that way?* Discussions also covered how we can research the experience of vulnerable groups.

In the community workshop this discussion moved seamlessly from setting research priorities towards ensuring the community’s information needs were adequately met. Whilst people understood the usefulness of conducting research, they saw raising awareness about air quality in the community of paramount importance. One attendee noted - *“make the evidence clear to people with straight-forward facts”* and someone else said this could be achieved by - *“displaying hard facts to make it difficult to ignore, (display) statistics all over the city with messages like turn your engines off outside schools”.*

We were advised that a good place to start raising awareness about air pollution is secondary schools as this is just the point in young people’s lives when they maybe considering driving lessons.

## The usefulness of the creative PPI method

Our approach paid heed to the advice offered by Baines and Regan de Bere, (2018) who emphasise the need to be creative when planning PPI activity. To quote them directly: *“do not rely on one method of communication; this is unlikely to be suitable for all those involved—be creative”* ^27^ (p.5). Relatedly, there is a growing body of literature exemplifying the value of art-based methods when used in health based research to achieve PPI objectives ^24, 28^ and there is important scholarly discussion in favour of art based approaches which praises the egalitarian ethos and the potential it holds to reduce alienation. Pool ^29^ makes this point fervently when he says: 

*“(T)hat doing research ‘on people’ can be an alienating process that despite the best intentions may not benefit participants or communities (…) and that the use of artistic methods, at worst, makes this process more open and accessible and, at best, offers viable and emancipatory forms of collective knowledge production”* ^29^ *(p.15)*

In the same document, Pool ^29^ makes use of bullet points to show the primary sources of motivation for why researchers turn to art based approaches. He states - a *“researcher’s desire to use artistic methods within their work is often underpinned by two fundamental assumptions:*

*1. The arts offer a space where participants in research are more willing to engage*.

*2. Artistic methods offer a potential to capture thoughts and ideas that are expressive, emergent and, to an extent, democratic”* ^29^ *(p11).*

We expand this by adding that artistic methods can reduce the heavy reliance on literacy skills as a prerequisite to engagement. This is important because low levels of literacy have been recorded in some neighbourhoods within the proposed CAZ area ^30^ and we know, through other published research, that areas with high rates of socio-economic deprivation tend to experience higher rates of illiteracy ^31^. Moreover, recent migratory patterns within some of the electoral wards in the proposed CAZ area indicate that many residents may not be fluent in English. Thus, by providing the options to colour and sketch onto the illustrated posters, along with the options to write and/or converse, we opened up opportunities for inclusion to many more people.

## Philosophical underpinning – pictures can become words

Alongside the improved inclusivity and creativity which art can offer, there is also a deeper philosophical appeal which draws a connection between language and pictures to the formation of knowledge as found in the writings of the philosopher Ludwig Wittgenstein ^32^. Whilst Wittgenstein’s ideas published a hundred years ago in the *Tractatus Logico Philosophicus* have gained popularity in many disciplines, they have not, as yet, penetrated the paradigm of inclusive and participatory approaches. This is somewhat surprising when we consider the topics of language and communication were the major catalyst for Wittgenstein’s rise to prominence, and as we shall see further in this paper, these are the same variables which continue to beset the PPI agenda. 

Before we explain the connection between Wittgenstein’s philosophy and our art-based PPI approach, it is worth elaborating on one of the unresolved challenges facing research professionals when communicating with members of the public. That is, researchers have a tendency to express ideas in ways that are familiar to their epistemological framework, which is often laden with esoteric and scientific terms, and can inadvertently lead to creating a communication divide between those doing the research and those whom the research is about ^33^. Whilst this issue appears to be a perennial problem affecting all aspects of the research process, the literature somehow leads one to suppose the problem is unique to the production of written materials associated with research. Consider the following quotes in support of this:

*Because many researchers write using technical, specialised language, particularly in scientific reports, writing plain English summaries can be challenging* ^33^ *(p.1)*

*Making a transition from the scientific to writing to or for the general public may fall outside a scientist’s comfort zone as explaining in common terms the complexities of science and research can be challenging* ^34^ *(p.578)*

Creating written material, which is easy-to-read, may well be challenging to produce but is valued by lay audiences ^35^ and for this reason has organically become mandated into the administrative processes surrounding research. ‘Plain English’ and ‘Lay summaries’ now occupy a central place in research documentation and appear in ethical approval forms, funding applications and in some cases peer reviewed journals ^34, 35^.

These are all steps in the right direction which can improve both inclusivity and accessibility. But the same principles have not found their way to include improved communication when engaging members of the public in a face-to-face conversational setting. Our search through the literature found no references which acknowledge and attempt to redress the language and communication gap through the use of creative methods when researchers come together with members of the public to discuss research ideas for PPI purposes. Where examples are available, these are for participation processes (i.e. data collection) and have been discussed favourably as being both inclusive and effective (see 36 as one example). Perhaps, there is a tacit assumption at play here which supposes that because PPI is done for the public, it would therefore naturally follow that any associated activity would have clear language and communication as a prerequisite condition. To stipulate this as essential might be considered stating the obvious. 

When assumptions contain misplaced ideas they can lead to inaccurate projections; for PPI purposes the assumption that clarity is achieved through verbal conversations because technical vocabulary may have been simplified, or because acronyms and jargon have been explained, overlooks the inherently complex nature of scientific research PPI groups are often presented with. Communicating complex ideas in a coherent way in the face-to-face setting is not without its challenges. The danger here is the pendulum can swing too far if researchers over-simplify the research plans by providing a minimalist ‘helicopter view’ about the subject matter. This quest for brevity and simplicity runs the risk of limiting lay members to contribute in a cursory way rather than a comprehensive manner. Such an approach could lead to the omission of important discussions about nuances and possible challenges that might prove crucial to the overall design.

If the primary aim of any PPI endeavour is to seek people’s experiential knowledge which can add value to the overall research plan ^37^ then skimming over and simplifying matters can lead to compromising this objective. This is not an easy dilemma to resolve because the alternative is to be broad and expansive. This can also run the risk of ‘information overload’ and can give rise to problems we mentioned at the start whereby complicated and esoteric concepts lead to confusion.

It is here where Wittgenstein’s ^32^ ideas prove useful. In the *Tractatus Logico-Philosophicus*, Wittgenstein gets to the nub of the communication problem by exploring the gulf between ‘what we mean’ when we express ideas and ‘what others understand’ from our expressions. Space and complexity preclude a detailed description of how the Tractatus operates (and to oversimplify it would be ironic considering the discussion); however, a hermeneutical interpretation of what Wittgenstein had to say about the solution to communication problems is briefly worth stating. Central to his philosophy is that – *“the logical picture can depict the world*” (p.29) because *“the picture contains the possibility of the state of affairs which it represents”* (p.29) in other words, the arrangement of names and descriptions we attach to ideas often take a logical pictorial form in the minds of people we communicate with and it is these pictorial forms which Wittgenstein states constitute a state of reality. Thus, the job of the person communicating should be to create a picture that reflects the reality they are describing and communicating. Wittgenstein thought of these pictures in a literal sense and went on to *say “what the picture represents is its sense*” (p.29) and *“(t)he picture depicts reality by representing a possibility of the existence and non-existence of atomic facts” (p.29).*

He did not say that everyone will draw the same reality as a necessary outcome from the communication and instead created ample room for the disagreements that might ensue. Equally, he stressed that a picture does not represent the truth about reality but saw it as a way to try and make sense of reality, as Wittgenstein ^32^ put it: “ *In order to discover whether the picture is true or false we must compare it with reality” (p.29).*

Whilst the above points have focussed solely on one segment of Wittgenstein’s many ideas and then further condensed this to a brief form; our PPI approach has shown adherence to this part of Wittgenstein’s philosophy. By creating and displaying images in a logical form and showing the different ways in which the Local Authority can attempt to reduce air pollution, we were painting a picture, both literally and figuratively, that gave our attendees the different states of reality for each of the CAZ options. By generating pictorial depictions at the outset, we were able to reduce the likelihood of any misunderstandings that may arise from unclear meanings about each of these different CAZ options. Achieving this level of clarity on this matter is important because the various permutations, along with the differential consequences of each of the CAZ options required a clear picture for people to offer their thoughts as part of the PPI activity. 

## Challenges

A sceptical viewpoint on using the art-based approaches could argue that this approach could lead to oversimplifying a complex range of problems by reducing them down to art form. The ‘means’ may be aesthetically pleasing and the approach may be creative but can the ‘end’ outcome ensure members of the public have been able to contribute in a meaningful way? This is an important challenge, especially when we consider that this method could lead to the exclusion of people who may not feel artistically confident. If such a scenario was to prevail then this would render art-based methods of PPI guilty of the same exclusion charges levelled at approaches which solely rely on the spoken or written word. 

For the above reasons, this is not a call to replace all other forms of PPI activities with art-based approaches, and nor should this imply all other PPI methods are tokenistic, but instead is a reflection of our experience to demonstrate that this approach has a place in the PPI toolkit which may suit certain activities for certain population groups. This fusion of art and research processes presents opportunities for knowledge production but also creates a challenging terrain which is summarised by Pool ^29^ in the following way:

*“An extensive process of cross-fertilization between research methods, art forms and artistic practice has created hybrid forms of research, art and knowledge production. However, the terrain is contested and problematic: process verses product, method opposed methodology, artists who see themselves as researchers, researchers who aspire to be artists, data versus evidence; the waters are muddy and there is no dry land.” (p.13)*

Fortunately, these muddy waters, for PPI purposes at least, provide a common and fertile ground for the exchange of information and meaningful dialogue. We found that the art-based PPI approaches correspond neatly to the philosophical traditions of Wittgenstein and in doing so ameliorates the problem of misunderstandings that can arise when two parties communicate with each other without reaching a mutual understanding. Pictures can help reduce this ambivalence by constructing a common understanding or a shared proposition because, as Wittgenstein ^32^ had famously said - *“we make to ourselves pictures of facts”* (p.28).

## Progress and next steps

Key points from the workshops have been reported to decision makers to help them formulate clean air plans by taking account of community perspectives. As mentioned earlier, this activity informed a qualitative research study to explore the views of seldom heard groups more formally ^17^, the findings from which influenced how policy makers in the Local Authority decided how to implement Bradford’s Clean Air Plan. For example, it supported them seeking higher levels of grant funding from central government to support taxi drivers and small/medium enterprises to upgrade vehicles, thus having a direct impact on the proposed delivery of the clean air zone. This PPI activity also informed extensive evaluation plans to explore the impact of the plan on air quality, health and economic outcomes for which external grant funding has been awarded. Details about our research and evaluation plans can be found via the ISRCTN registration website. 

In progressing this further, the role of community members will remain central to our research efforts as we will continue to capitalise on their expertise by involving them in a number of ways. This will include activities such as advising academic partners on research questions, data collection methods, analysis and dissemination plans. In line with the World Health Organisation (WHO) guidelines ^38^, our approach will help create harmony between implementation and research plans and community perspectives and priorities. WHO ^38^ guidance states *“cities should demonstrate increased public participation in the decision making processes that affect health in the city, thereby contributing to the empowerment of local people”* (p.6).

## Concluding remarks

The art-based community workshops have bestowed us with two important insights. Firstly, a vital awareness about the potential impact of the various CAZ options on communities; and secondly a realisation about the type of questions we should focus on as part of our research and evaluation plans.

This paper has focussed a spotlight on the problematic gaps of language and communication which can create a divide between the researchers and communities when delivering PPI activities. Much of this divide stems from the intimate familiarity professionals hold about the research subject, coupled with the assumption that the target audience will be in a position to contribute their thoughts after an exchange of rudimentary information. We have shown how depicting the matter into image form, using art-based approaches, can be an effective way to bridge this void because it can create a shared reality. Wittgenstein words - ‘an image can reach out to reality’ ^32^ seem to have a ready application for patient and public involvement.

## Data availability

No data are associated with this article.
