# Supplementary material for: The art of Patient and Public Involvement: exploring ways to research and reduce air pollution through art-based community workshops – a reflective paper
Source: Wellcome Open Res. 2022 May 26;7:162. [Version 1] doi: 10.12688/wellcomeopenres.17886.1 (PMC9396111; doi:10.12688/wellcomeopenres.17886.1)
Supplement: Peer review history

# Peer review: Reviewer response for version 1

**DOI:** 10.21956/wellcomeopenres.19816.r50850

**Published:** 2022-08-22

**Authors:** Anna Cronin de Chavez

**Affiliations:** 1 Global Health and Development, London School of Hygiene & Tropical Medicine, London, UK

This is an essential paper providing experiential knowledge of using an arts based method for PPI that can contribute to more effective PPI in future studies in general, not just in the field of air pollution or in the geographic setting in Bradford. As the paper highlights, to give more importance to PPI, it can be helpful to share how it is done and reflect on experiences. So thank you for giving me the opportunity to review this article on this under-looked topic. 

 A few more minor comments to improve details in the text are suggested below but a more major comment would be to make a stronger analysis and argument about coproduction in intervention design and research, the wider landscape, political considerations and how PPI fits into the c0-production landscape. It would also be useful to expand on this explanation in the ‘inherent challenges of PPI’ section, and set it in the landscape of co-production (draw on wider literature) – what is coproduction? What forms can it take? How does PPI and this activity fit into this picture? Any reflections on how suitable it is? How could have this have been done differently? You could also pick up on the motivational factors, and limits. For example the community may want to have more choice around research and interventions but this rarely happens, funders may be quite keen on funding projects with PPI included in the plans but be happy with a nominal treatment when agreeing funding and then have no requirements to ensure it happens in practice. So where are the motivations and priorities coming from? These issues can be discussed in the sections 'the Inherent challenges of PPI’ and ‘the Usefulness of the creative PPI methods’ and ‘Philosophical underpinning’.

 How does PPI feedback translate into decision making? A section on data analysis is missing. Also the authors would do well to provide a stronger argument as to why an arts-based approach is an advantage? For who? How? Not all readers will initially appreciate why. There is a long section on Wittenstein which is perhaps disproportionate, this part could be reduced and more attention could be paid to coproduction in general as suggested above. The authors also could have mentioned broader limitations in participation – e.g. who was and was not able to participate due to not being able to access them or they were unable to attend meetings and venues? This would fit with the consideration of co-design in general – who is consulted and how?

 Title:

- Include coproduction in the title so you will get more views and citations as this is central to the article

Abstract:

- Be more specific about who the air pollution (AP) and charges are relevant to – population? Setting? Problem?

Background:

- First sentence – just cite the article or it looks like you are basing your study just on this article's evidence.
- 64k deaths in the UK? Global?
- Specify if AP is higher in urban deprived communities (not rural).
- The 2 ^nd^ para, 2 ^nd^ sentence is not an additional element but an explanation so frame this as such.
- 3 ^rd^ para, 2 ^nd^ sentence add years of policies and add equivalent of ‘local authority’ (eg municipalities) for non UK readers. 

Why do community voices matter?

- Explain acronyms and when talking of clean air zones and specify what area is being talked about.

The inherent challenges of PPI:

- The points here are very important and so could be highlighted in the abstract more.

Our art-based PPI method:

- Describe the method as it is currently unclear. And why have you chosen this?

Ethics:

- The last sentence perhaps belongs in methods.

Invitation, attendence and venues:

- Give more detail on demographics and consider the power balances within the groups.

Workshop content:

- How were the posters used?

Format:

- More details about how many and who were in each workshop please.

Key highlights from the workshops:

- More decisions around what difference it makes to the decision making if the community are consulted in this stage of the design and what could be done differently in the future? How representative is this of many PPI projects? Why? What limitations may be expected for an ideal scenario in future projects?

Figs. 1 & 2:

- Great graphic but be aware that the writing may be too small to read in a PDF version – so consider accessibility in general throughout the document. See more on improving accessibility in documents here (for this and future work) https://daisy.org/

Progress and next steps:

- Who are the ‘decision makers’?

Does the article adequately reference differing views and opinions?

Yes

Are all factual statements correct, and are statements and arguments made adequately supported by citations?

Partly

Is the Open Letter written in accessible language?

Yes

Where applicable, are recommendations and next steps explained clearly for others to follow?

Partly

Is the rationale for the Open Letter provided in sufficient detail?

Yes

Reviewer Expertise:

Public health, global health and medical anthropology

I confirm that I have read this submission and believe that I have an appropriate level of expertise to confirm that it is of an acceptable scientific standard, however I have significant reservations, as outlined above.

# Peer review: Reviewer response for version 1

**DOI:** 10.21956/wellcomeopenres.19816.r50851

**Published:** 2022-06-13

**Authors:** John Hiley

**Affiliations:** 1 Department of Research & Knowledge Services, Bradford District NHS Care Trust, Shipley, UK

This a welcome and timely paper, addressing the perennial issues of valid and meaningful PPI in the formation of public policy and academic research.

 Applying novel methods to what can be a dry and difficult process for both members of PPI panels or groups, and also for those attempting to elicit PPI in their work, is refreshing, and I applaud these efforts.

 The identification of language barriers is an important one. Not only in a 'native' spoken language however, but very much in the technical 'restricted code' that workers/experts in their own particular fields tend to unconsciously fall into. This comes on top of scientific concepts that may be unfamiliar to the members of any PPI group that truly represents those members of the public whose voices are, so often, not only unheard, but also unsolicited.

 As such, it would be useful for the reader to have greater detail of how the project progressed, what form of responses were received (perhaps with some imagery), and how these pieces of work were themselves interpreted by the authors. How easily transferable would these skills be to a 'jobbing' researcher? I accept that this may be the subject of writings elsewhere, or perhaps limited by the space here.

 It would be interesting for the team to also discuss the potential for further work looking at both the issues, both positive and less so, of recruiting people already active in their communities, and of the value of investment by researchers in breaking down their 'restricted code' and facilitating understanding of key issues for the PPI members, as part of their investment in the effectiveness of the PPI process. (There is also a little irony that the section on philosophical underpinnings, itself disappears into the language of academic philosophy, albeit briefly). 

 This otherwise compelling and very readable piece, opens the door to further discussions surrounding more effective PPI methodology than plonking individuals into a room of academics, clinicians or the like, where the well intentioned atmosphere may serve only to intimidate the expression of the views the research team so clearly want.

Does the article adequately reference differing views and opinions?

Yes

Are all factual statements correct, and are statements and arguments made adequately supported by citations?

Yes

Is the Open Letter written in accessible language?

Partly

Where applicable, are recommendations and next steps explained clearly for others to follow?

Yes

Is the rationale for the Open Letter provided in sufficient detail?

Yes

Reviewer Expertise:

Mental health and dementias, including experiences of PPI and the training of service user researchers.

I confirm that I have read this submission and believe that I have an appropriate level of expertise to confirm that it is of an acceptable scientific standard.

# Peer review: Reviewer response for version 1

**DOI:** 10.21956/wellcomeopenres.19816.r50847

**Published:** 2022-06-09

**Authors:** Jane Montague

**Affiliations:** 1 Faculty of Health Studies, University of Bradford, Bradford, UK

This is an interesting and engaging article that seeks to outline and discuss an art based PPI approach to exploring air pollution reduction with members of the public living in air pollution hotspots within the city of Bradford. I very much enjoyed reading it.

 The first half focuses on the context, the problem and the art based PPI activity, and the second is a thoughtful and informed reflective piece on the use of language and communication in the research process, particularly in using PPI and involving communities.

 Both the PPI activity and the reflection are innovative in their approaches and both have much to offer other researchers in their work with communities. This is a key strength of the article, indeed of the endeavour generally. The authors are also mindful to reflect on the limitations of the approach, however exciting it may appear, and to address the pitfalls of meaning and nuance when there are multiple understandings.

 I do not think this is an easy task: to marry such a diverse content (one practical, the other philosophical) and there is a risk that it could read as two separate papers. It might be that the authors work on integrating it a little more successfully. I think this could be done by paying attention to the abstract, introduction and conclusion.

 For example, I felt that the abstract did not really reflect the strength and innovativeness of the paper. It is only in the final paragraph that this is conveyed. The information in the first two paragraphs of the abstract felt like it should more be part of an introduction: this seemed missing in the main body. The article begins with the background on air pollution rather than setting the scene for the complete piece of work that the article comprises. I would amend the abstract to reflect more overtly the article - it is so much more than is currently presented in it - and I would include something at the beginning of the article that offers the reader an overview of the two aspects of the whole. 

 I am also not sure that the conclusion is successful. It could be much more coherent; the two paragraphs feel rather disparate (see comments above; perhaps in addressing the introduction, this would lead to a more companionable conclusion).

 Finally, I wondered in the discussion of the community/ies involved (sections: 'Invitation, attendance and venues'  and 'usefulness of the creative PPI method') whether a little more could be said about the identities of those communities There is a reference to recent migration and fluency of the English language, as well as literacy but it is fleeting. Equally socioeconomic status is referred to but not specifically related to the community/ies in question. The authors may have decided these were less significant factors for coverage/discussion but given that the heart of the reflective discussion is on language and communication I feel they are important.

 Overall however I would like to thank the authors for their very interesting article and wish them well in the research that arises from it. 

 Note: I have 'approved with reservations' but the statement that accompanies that choice is much less complimentary than I would like. I would say some changes are needed but not 'significant' and it is not without merit.

Does the article adequately reference differing views and opinions?

Yes

Are all factual statements correct, and are statements and arguments made adequately supported by citations?

Yes

Is the Open Letter written in accessible language?

Yes

Where applicable, are recommendations and next steps explained clearly for others to follow?

Not applicable

Is the rationale for the Open Letter provided in sufficient detail?

Yes

Reviewer Expertise:

Public Health (refugee and asylum health); Digital Health (innovation and technology usage in healthcare)

I confirm that I have read this submission and believe that I have an appropriate level of expertise to confirm that it is of an acceptable scientific standard, however I have significant reservations, as outlined above.
